# Can the municipal social deprivation index influence the time trend of the leprosy detection rate?

**DOI:** 10.1590/0037-8682-0228-2020

**Published:** 2020-11-13

**Authors:** Carlos Dornels Freire de Souza, Victor Santana Santos, Joilda Silva Nery, Tânia Rita Moreno de Oliveira Fernandes, Mônica de Avelar Figueiredo Mafra Magalhães

**Affiliations:** 1 Universidade Federal de Alagoas, Núcleo de Estudos em Medicina Social e Preventiva, Departamento de Medicina, Arapiraca, AL, Brasil.; 2 Universidade Federal de Alagoas, Núcleo de Epidemiologia e Saúde Pública, Departamento de Enfermagem, Arapiraca, AL, Brasil.; 3 Universidade Federal da Bahia, Instituto de Saúde Coletiva, Salvador, BA, Brasil.; 4 Universidade Federal do Vale do São Francisco, Colegiado de Medicina, Petrolina, PE, Brasil.; 5 Fundação Oswaldo Cruz, Instituto de Comunicação e Informação Científica em Saúde, Rio de Janeiro, RJ, Brasil.

Dear Editor:

Leprosy is an infectious disease caused by *Mycobacterium leprae* that affects the skin and peripheral nerves and may result in physical disabilities and/or deformities^1^, which are associated with functional limitation, social isolation, stigma, and low quality of life^1^.

Although the burden of leprosy has reduced in recent decades, in 2017, more than 210,000 new cases have been reported in 150 countries, resulting in a global detection coefficient of 2.77/100,000 population^2^. In Brazil, more than 28,000 cases are registered annually^3^. 

Because it is considered a disease affecting low-income populations, the process of leprosy disease is potentially determined by the social context of the individuals^4^ ^,^ ^5^. For this purpose, the Social Needs Index can be used^6^. The objective of our study was to analyze the temporal trends in the detection rate of new leprosy cases in the general population according to the stratum of the municipal Social Needs Index (SNI) in Bahia, Brazil, from 2001 to 2015.

This was an ecological study involving all new cases of leprosy diagnosed in residents in Bahia, Brazil, from 2001 to 2015. The investigation process occurred in three stages:

*Step 1* - Calculation of the detection coefficient of new leprosy cases in the general population, based on the number of cases obtained from the National Information System for Notifiable Diseases (SINAN, in Portuguese) and population data from the Brazilian Institute of Geography and Statistics (IBGE). Crude and smoothed indicators were calculated by the local empirical Bayesian model. The smoothing was needed to reduce random fluctuation of the data.

*Step 2* - Obtaining the Social Needs Index (SNI): The SNI was elaborated according to the methodology proposed by UNICEF^6^. The SNI involved four variables: 1) Economic and Social Performance-Economy and Finance Index (IPESE-EF), (2) average monthly value of per capita income (RENDAPERCAPIT), (3) proportion of extremely poor (%EXTRPOBRES), and (4) number of households with density larger than three persons per room (DOM3PPDOR). These variables were selected from previous studies in which these indicators were associated with the dynamics of leprosy transmission in Bahia state^4^ ^,^ ^7^. After the calculation, the municipalities were classified into quartiles: low SNI (0.142 to 0.259), medium SNI (0.260 to 0.369), high SNI (0.370 to 0.479), and very high SNI (0.480 to 0.699).

*Step 3* - For the trend analysis, we used the Joinpoint regression model^8^. Trends were classified as increasing, decreasing, or stable. The annual percentage change (APC) was also obtained, considering a 95% confidence interval (95% CI) and significance level of 5%.

The project was approved by the Human Research Ethics Committee of the Federal University of Alagoas: Protocol No. 2.212.723, of August 10, 2017.

Between 2001 and 2015, 42,227 new leprosy cases were registered in Bahia state. The detection coefficient of new cases was 26.61/100,000 population in 2001, and it declined to 14.7/100,000 population in 2016, classifying Bahia state as highly endemic (between 10.0 and 19.9 new cases/100,000 population). 

A total of 252 (27.31%) municipalities were classified as very high SNI. The low and medium SNI showed a decreasing trend of the detection coefficient between 2005 and 2004, being high in the group with low SNI (APC -9.2% for the crude rate and APC -8.2% for the smoothed rate). Considering the time series (2001-2015), only the low SNI group showed a significantly decreasing trend (APC -2.9% for the crude rate and APC -2.6% for the smoothed) (Table 1).

**TABLE 1:** Trend of detection coefficients of new leprosy cases in the general population, according to the Social Needs Index (SNI) stratum in Bahia state, Northeast Brazil, 2001-2015.

Social Needs stratum	Municipalities n (%)	Period	APC (95% CI)	P-value	Trend
Coefficient of detection of new leprosy cases - Crude indicator					
Low	12 (2.88)	2001-2005	14.8 (4.2 to 26.5)	<0.001	Increasing
		2005-2015	-9.2 (-11.4 to -7.0)	<0.001	Decreasing
		2001-2015	-2.9 (5.7 to -0.1)	<0.001	Decreasing
Medium	39 (9.35)	2001-2004	15.0 (-7.8 to 43.3)	0.25	Stable
		2004-2015	-4.6 (-7.4 to -1.7)	<0.001	Decreasing
		2001-2015	-0.7 (-5.2 to 4.0)	0.88	Stable
High	114 (27.34)	2001-2006	15.3 (7.1 to 24.1)	<0.001	Increasing
		2006-2019	-17.0 (-40.4 to 15.4)	0.21	Stable
		2009-2015	-0.5 (-5.9 to 15.4)	0.90	Stable
		2001-2015	0.9 (-5.5 to 7.7)	0.81	Stable
Very high	252 (27.31)	2001-2003	35.5 (-11.5 to 107.4)	0.11	Stable
		2003-2015	-4.9 (-7.3 to 2.5)	0.43	Stable
		2001-2015	0.0 (-5.5 to 5.9)	1.0	Stable
Coefficient of detection of new leprosy cases - Smoothed indicator					
Low	12 (2.88)	2004-2004	20.9 (6.3 to 37.5)	<0.001	Increasing
		2004-2015	-8.2 (-9.7 to -6.5)	<0.001	Decreasing
		2001-2015	-2.6 (-5.2 to -0.1)	<0.001	Decreasing
Medium	39 (9.35)	2001-2014	13.3 (-4.1 to 33.8)	0.13	Stable
		2004-2015	-4.3 (-6.4 to -2.1)	<0.001	Decreasing
		2001-2015	-0.8 (-4.2 to 2.8)	0.77	Stable
High	114 (27.34)	2001-2005	13.5 (2.5 to 25.7)	<0.001	Decreasing
		2005-2015	-5.6 (-8.0 to -3.2)	<0.001	Decreasing
		2001-2015	-0.5 (-3.5 to 2.5)	0.71	Stable
Very high	252 (27.31)	2001-2004	18.7 (-2.0 to 43.8)	0.13	Stable
		2004-2015	-5,2 (-7,6 to 2.7)	0.32	Stable
		2001-2015	-0.5 (-4.4 to 3.6)	0.80	Stable

Legends: **APC**, Annual Percent Change.

Several investigations have shown that the risk of leprosy is high among people living in poor living conditions^4^ ^,^ ^5^ ^,^ ^7^ ^,^ ^9^. Income inequality, social vulnerability, poverty, poor housing conditions, poor diet, and low educational level are considered social determinants related to disease transmission^4^ ^,^ ^5^ ^,^ ^7^ ^,^ ^9^. However, poor conditions may inhibit the early diagnosis, thus increasing the hidden prevalence, which justifies the low coefficients observed in the strata of higher SNI.

General social improvements may be able to promote a reduction in the burden of leprosy. Investigations on the effects of policies on the magnitude of leprosy, for example, showed that municipalities with the largest coverage of *Bolsa Família* had the largest reductions in disease detection coefficients^10^. Such public policies have also reached the most vulnerable municipalities, and this may justify the reduction in the rate of leprosy detection in the medium and high social vulnerability strata, as observed in this study.

Finally, we recommend that in endemic areas, reducing social deprivation may result in disruption of the disease transmission chain and subsequent decline in the coefficient.
